# The Association of Postpartum Maternal Mental Health With Breastfeeding Status of Mothers: A Case-Control Study

**DOI:** 10.5812/ircmj.14839

**Published:** 2014-03-05

**Authors:** Fatemeh Assarian, Alireza Moravveji, Hamideh Ghaffarian, Reihaneh Eslamian, Fatemeh Atoof

**Affiliations:** 1 Department of Psychiatry, Kashan University of Medical Sciences, Kashan, IR Iran; 2 Social Determinants of Health Research Center, Department of Community Medicine, Kashan University of Medical Sciences, Kashan, IR Iran; 3 Deputy of Health, Kashan University of Medical Sciences, Kashan, IR Iran; 4 Student Research Committee, Kashan University of Medical Sciences, Kashan, IR Iran; 5 Department of Epidemiology and Biostatistics, Tehran University of Medical Sciences, Tehran, IR Iran

**Keywords:** Breast feeding, Maternal Welfare, Mental Health, Iran

## Abstract

**Background:**

Maternal health status might have an important effect on breastfeeding, growth, and general health of the infants.

**Objectives:**

This study was conducted to assess the association between maternal mental health and breastfeeding status of mothers in Kashan province.

**Patients and Methods:**

This case-control study was conducted on 458 mothers in two groups of unsuccessful breastfeeding (case) and successful breastfeeding (control) attending Kashan province health clinics. In this study, the GHQ questionnaire and clinical interview were employed to collect data. The data were statistically analyzed using Chi-square and Fisher’s exact tests.

**Results:**

It was found that mothers of the case group had a greater susceptibility to depression than those of the control group, that is, breastfeeding status was directly associated with susceptibility to depression (P = 0.001, OR = 5.48). Furthermore, there was a significant association between basic characteristics such as maternal occupational status (P = 0.04) or their educations (P = 0.006) with breastfeeding. Besides, clinical interview revealed that mixed depression and anxiety disorder was the most prevalent type of psychological disorder in the case group.

**Conclusions:**

Screening depression during pregnancy and postpartum period appeared to be necessary and it should be incorporated into prenatal and postnatal care due to its influence on mothers’ successful breastfeeding.

## 1. Background

It is now widely accepted that to have the optimal outcomes for both mother and infant, breastfeeding is the ideal way of feeding infants. Breastfeeding not only is the best source of energy and providing the optimum source of nutrition for the growth of infant, but also guarantees good protection from infectious diseases and allergies through favorable physical, neurological, and cognitive development (1-3). An increased risk or morbidity and mortality from respiratory tract infections, atopic dermatitis, childhood asthma, type II diabetes, obesity, and sudden infant death syndrome endanger babies who are not breastfed (4, 5). A longer duration and intensity of breastfeeding maximizes the health benefits of breast milk (6). It is also well known that exclusive breastfeeding for the first six months of life ensures the best outcomes for both infant and mother (1, 2).

In a recent study, it was indicated that psychosocial factors would predict exclusive breastfeeding duration more effectively than demographic factors (7). Breastfeeding cessation was influenced by pre-partum levels of anxiety and depression, and in turn, increased the risk of postpartum anxiety and depression (8, 9). In another study, an inverse association between breastfeeding frequency and maternal anxiety level was found (10). Breastfeeding cessation is a risk factor for increased anxiety and depression (8). Thus, it appears that there is a mutual association between maternal mental health and exclusive breastfeeding. Mothers with exclusive breastfeeding had higher breastfeeding self-efficacy (11). Keeping all the aforementioned advantages of exclusive breastfeeding in mind, it is worth noting that at present, less than 40% of infants under six months of age are exclusively breastfed worldwide (12).

In Iran, only 65.8% of mothers would continue to exclusively breastfeed their infants until four months of age. According to the results of IMES project conducted in Kashan in 2005, only 50% of infants younger than four months and 35% of infants younger than six months were exclusively breastfed. Early cessation of breastfeeding was ascribed to factors such as mother belief in her milk insufficiency, infant agitation and crying, friends and family recommendations, and physician prescription due to mother or child disease. Previous breastfeeding experience, particular personal criteria (e.g. indifference, acceptance of female role, coping well with life events, affection and attachment for child and child nurturing), environment, culture, and child health status influence mother’s decision to breastfeed her child (13). Mothers who display symptoms of anxiety, stress, and depression are prone to breastfeeding failure (14). In addition, a greater possibility to cease breastfeeding in mothers with a higher degree of depression and anxiety exists (15).

Maternal mental health has major impacts on breastfeeding, growth and health of the child. Since no similar study had been conducted in Kashan province and the breastfeeding cessation rate was high, we aimed to assess the association between maternal mental health and breastfeeding status and to find the underlying psychological disorder. It would enable us to promote the health of the children, mothers, families, and society by adjusting and controlling the factors underlying breastfeeding cessation.

## 2. Objectives

This study was conducted to assess the association between maternal mental health and breastfeeding status of mothers in Kashan province.

## 3. Patients and Methods

This case-contrl study with convenient sampling strategy was conducted on a total of 458 mothers with infants aged younger than one-year-old who were recruited from Health and Treatment Clinics in Kashan/Iran. The sample size was determined by Using the comparison of two proportions formula and the results of similar studies with the risk of depression in mothers with successful (control) and unsuccessful (case) breastfeeding being 39% and 52%, respectively, a type I error of 5%, and a power of 80%. Thus, the sample size was estimated as 229 in each group (458 totally).

Mothers with an infant younger than one year of age during her health care consultation were included in the study. Mothers with the following conditions were included in the unsuccessful breastfeeding (case) group: already initiated bottle-feeding, to be incapable of breastfeeding due to her own dysphoric mood, malaise, the infant’s crying(restlessness), or growth retardation, or qualified for getting formula and referred to the Breastfeeding Promotion Commission . On the contrary, a mother with successful breastfeeding from the same health center who was otherwise matched to her counterpart in the case group (i.e. according to infant’s age, mother’s age, occupation, and education) was included as a control. For case group, participants with the following condition were excluded from the study: an established psychiatric disorder of the mother, history of taking drugs contraindicated to lactation, child adoption, mother’s death, and multiple birth. After identifying the participants and obtaining written consent from them, they were asked to complete the demographic questionnaire and GHQ-28.

The GHQ was first developed by Goldberg in 1972 and has been widely used to screen emotional distress and psychiatric morbidity. Available in a variety of versions of 12, 28, 30 and 60, the 28-item version is the most widely used. The GHQ-28 is divided into four subscales including somatic symptoms (items 1-7), anxiety/insomnia (items 8-14), social dysfunction (items 15-21), and severe depression (items 22-27). The scoring method used here was based on Likert method in which each response is scored from zero to three with a total possible score ranging from zero to 84. The cut-off point for each subscale and the entire questionnaire is 14 and 23, respectively. The validity and reliability of GHQ-28 was estimated in different studies to be 90% (16). After determining mothers with scores above the cut-off point, they were clinically interviewed based on DSM-IV TR criteria.

### 3.1. Ethical Consideration

The study was approved by the Ethic Committee of Kashan University of Medical Sciences. All ethical issue such as informed consent, plagiarism, double publication and/or submission were considered. The respondents were anonymous and participated willingly in this study.

### 3.2. Statistical Analysis

To assess the association of breastfeeding status with independent variables, data analysis was performed by the SPSS version 16 (SPSS Inc., Chicago, Illinois, USA) using descriptive statistics (absolute frequency, relative frequency, mean, standard deviation) and OR, Chi-square test or Fisher’s exact test if applicable.

## 4. Results

In this study, mothers were classified according to successful and unsuccessful breastfeeding into two groups of case (227 mothers who both breastfed and bottle-fed their infants) and control (231 mothers who breastfed their infants exclusively). Among the 458 mothers studied, 345 were homemakers (75.3%) and 113 were employed (24.7%); the proportion of homemaker mothers in the case group was higher than that of control group (79.7% and 71%, respectively). Odds ratio with regard to breastfeeding status was 1.61 (Table 1).

With regard to educational status, the majority of mothers had elementary school level (27.3%) compared with high school level (41.1%) in the case and control groups, respectively (Table 2).

In the case group, 114 mothers had poor mental health while 113 mothers were completely healthy. In control group, 82 (35.5%) and 149 (64.5%) mothers had poor mental health or were completely healthy, respectively. A significant association between mother’s mental health and breastfeeding status existed (P = 0.001). Moreover, an odds ratio of 1.83 suggested that mothers with unsuccessful breastfeeding were 1.83 times as susceptible to a psychological disorder as mothers with successful breastfeeding. Based on GHQ-28, there was no significant association between anxiety, social function, and somatic complaint with breastfeeding status (Table 3). However, there was a statistically significant association between depression sub-scales and breastfeeding status, thereby making unsuccessful mothers in breastfeeding 5.48 times as susceptible to depression as successful mothers.

Among 196 mothers studied, who were suspected to have mental disorder based on GHQ-28, 88 were diagnosed with a mental disorder based on clinical interview (Table 4). Furthermore, in the course of clinical interview, depression and anxiety were diagnosed as the most frequent disorder among 61 (36.6%) mothers of the case group while 27 (27.5%) mothers of the control group had depression and anxiety (Table 5).

**Table 1.** Occupation of Mothers in Case and Control Groups ^a,b^

Groups	Homemaker	Employed	Total	Statistical Test
**Case**	181 (79.7)	46 (20.3)	227 (100)	P = 0.03
**Control**	164 (71)	67 (29)	231 (100)	CI = 1.04-2.47
**Total**	345 (75.3)	113 (24.7)	458 (100)	OR = 1.61

^a^ Abbreviations: CI, confidence interval; OR, odds ratio.

^b^ Data are presented as No. (%).

**Table 2.** Educational Status of Mothers in Case and Control Groups ^a,b^

	Case	Control	Total
**Illiterate**	10 (4.4)	3 (1.3)	13 (2.8)
**Elementary school**	62 (27.3)	46 (19.9)	108 (23.6)
**Middle school**	38 (16.7)	45 (19.5)	83 (18.1)
**High school**	61 (26.9)	95 (41.1)	156 (34.1)
**Associate’s degree**	25 (11)	16 (6.9)	41 (9)
**Bachelor’s** **and higher degrees**	31 (13.7)	26 (11.3)	57 (12.4)
**Total**	227 (100)	231 (100)	458 (100)

^a^ Data are presented as No. (%).

^b^ P value = 0.006.

**Table 3.** GHQ Subscales in Case and Control Groups ^a^

	Case	Control	P Value
**Somatic complaint**			0.2
Yes	18 (7.9)	12 (5.2)	
no	209 (92.1)	219 (94.8)	
**Social function**			0.08
Yes	16 (7)	8 (3.5)	
no	211 (93)	223 (96.5)	
**Anxiety**			0.06
Yes	32 (14.1)	20 (8.7)	
no	195 (8.9)	211 (91.3)	
**Depression**			0.001
Yes	20 (8.8)	4 (1.7)	
no	207 (9.2)	227 (98.3)	

^a^ Data are presented as No. (%).

**Table 4.** Mental Disorders in Mothers Based on Clinical Interview ^a,b^

	Case	Control	Total
**Mental disorder**	10 (14.1)	13 (32.5)	23 (20.7)
**No mental disorder**	61 (85.9)	27 (67.5)	88 (79.3)
**Total**	71 (100)	231 (100)	111 (100)

^a^ Data are presented as No. (%).

^b^ P value = 0.022.

**Table 5.** Different Types of Mental Disorders in Mothers based on Clinical Interview ^a,b,c^

	Case	Control	Total
**No mental disorder**	10 (14.1)	13 (32.5)	23 (20.7)
**Depression**	13 (18.3)	11 (27.5)	24 (21.6)
**Anxiety**	6 (8.5)	9 (22.5)	15 (13.5)
**Depression and anxiety**	26 (36.6)	3 (7.5)	29 (26.1)
**Phobia**	8 (11.3)	1 (2.5)	9 (8.1)
**OCD**	8 (11.3)	3 (7.5)	11 (9.9)
**Total**	71 (100)	40 (100)	111 (100)

^a^ Abbreviation: OCD, obsessive compulsive disorders.

^b^ Data are presented as No. (%).

^c^ P value = 0.001.

## 5. Discussion

The current study was conducted to determine the breastfeeding status and mental health of mothers attending health clinics of Kashan. According to our results, 50.2% of mothers with unsuccessful breastfeeding and 35.5% of controls did not have a good mental health with a significant difference between two groups (P = 0.001). In a study by Mezzacapa, it was demonstrated that mothers who breastfed their infants had a better mental and physical health in comparison with those who bottle-fed their infants (17). Furthermore, Kendall-Tackett showed that breastfeeding reduced mothers’ negative mood (18). In another study conducted on Bangladeshi and Pakistani women in southwest of England by Noor, the mean mood score of mothers with exclusive breastfeeding was greater than that of mothers with either mixed-breastfeeding or exclusively bottle-feeding (19). As can be seen, there is a consensus among the findings of aforementioned studies and the present study.

In this study, mothers with unsuccessful breastfeeding obtained higher scores in the depression subscale than those of mothers with successful breastfeeding (P = 0.001). Moreover, based on clinical interview, mixed depression and anxiety disorder and then depressive disorders were higher in these mothers than in mothers of the control group. Such results are confirmed by Durheim study that indicated mothers without exclusive breastfeeding were likely to suffer from sleep disorders more frequently and are prone to a greater risk of depression (20). In addition, Taveras, Henderson and Akman in their studies showed that ceasing breastfeeding during the first trimester was associated with mother’s depression and that postpartum depression heightened the risk of ceasing breastfeeding as well (15, 21, 22). Groer studies would further corroborate the results of the present study as they suggested that mothers who exclusively breastfed their infants would be less responsive to stress and might have a lower depression index (23). Nevertheless, in a study conducted on 300 mothers by Yasemi, the average score of depression among mothers of the exclusive breastfeeding group were lower while not significant. This could be ascribed to a smaller sample size and using a different measurement tool like Beck Inventory (24).

In our study, there was no significant difference between symptoms of anxiety in mothers with mixed breastfeeding and mothers of the control group, which is consistent with the findings of Ackman study (23). However, this is in contrast to the results obtained by Yasemi indicating a significant association between anxiety and feeding method, that is, mothers with mixed breastfeeding or those who ceased breastfeeding were prone to a higher anxiety than those with exclusive breastfeeding (25). Experiences such as breastfeeding, that facilitate the reciprocal interaction between mothers and infant by providing the mother with a stronger sense of knowing her infant, might decrease maternal anxiety by helping the mother to develop confidence in her assessment of the infant's needs (25).

A review of literature revealed that psychosocial factors played a prominent role in maintaining exclusive breastfeeding during the first six months. Self-efficacy, postpartum depression, and tendency towards breastfeeding are important predictors of exclusive breastfeeding (26). Breastfeeding has a major role in the postpartum mental health of mothers; Groer showed that breastfeeding had beneficial psychoneuroimmunologic effects on mothers (27). Lowering stress level, breastfeeding keeps mothers’ favorable mood and contributes to a greater comfort, decreased response to stress, and rewarded maternal breastfeeding behavior. The more the level of stress is reduced, the less the response of the sympathetic nervous system will be, which in turn guarantees a lower risk of depression (18). Overall, there might be a noteworthy causal association between maternal mental health and the feeding method selected by mother (i.e., breastfeeding, mixed breastfeeding, or bottle-feeding), the direction of which is not yet well established. Given the importance of such an association irrespective of its direction, it would sound eminently reasonable to incorporated maternal mental health into the agenda of community-based nutrition programs.

### 5.1. Strengths and Weaknesses of the Study

This study was done for the first time in Kashan and we used clinical interview to confirm the results of questionnaires; however, we did not assess other psychological factors that could influence on exclusive breastfeeding. The results of this study are not generalizable to other cultural groups.
